# Clinical Effects of Baduanjin Qigong Exercise on Cancer Patients: A Systematic Review and Meta-Analysis on Randomized Controlled Trials

**DOI:** 10.1155/2021/6651238

**Published:** 2021-04-08

**Authors:** Chi-Chun Kuo, Chiao-Chen Wang, Wei-Lun Chang, Tzu-Ching Liao, Pei-En Chen, Tao-Hsin Tung

**Affiliations:** ^1^Department of Public Health, Kaohsiung Medical University, Kaohsiung, Taiwan, China; ^2^Institute of Health Policy and Management, National Taiwan University, Taipei, Taiwan, China; ^3^Taiwan Association of Health Industry Management and Development, Taiwan, China; ^4^Evidence-Based Medicine Center, Taizhou Hospital of Zhejiang Province Affiliated to Wenzhou Medical University, Linhai, Zhejiang, China

## Abstract

**Objective:**

Baduanjin is a traditional Chinese Qigong exercise for health improvement. However, a few studies were examining the association between Baduanjin Qigong exercise and cancer patients. This study is conducted to explore the clinical effects of the Baduanjin Qigong exercise among cancer patients.

**Methods:**

We conducted a systematic review and meta-analysis using randomized controlled trials to assess the effects of the Baduanjin Qigong exercise on cancer patients. We searched Cochrane Library, PubMed, Embase, and Airiti Library for all relevant studies from inception through December 31, 2020, without language limitations. Two authors independently screened selected studies, assessed the quality of included studies, and extracted information. Any disagreement was discussed with a third senior author. Summary estimates were obtained using meta-analysis with the random effects model.

**Results:**

Among the fourteen articles involved in the systematic review, ten studies were included in the meta-analysis. Cancer patients with moderate-severe cancer-related fatigue were significantly less in the Baduanjin group compared with the control group (odds ratio = 0.27; 95% confidence interval (CI) [0.17, 0.42]). Three studies used the questionnaire of Functional Assessment of Cancer Therapy-Breast Cancer (FACT-B) in the assessment of quality of life, and two used the European Organization for Research and Treatment of Cancer Quality of Life Questionnaire (EORTC QLQ-C30). For FACT-B, the Baduanjin group scored significantly higher than the control group (mean difference = 11.04, 95% CI [9.56, 12.53]). For EORTC QLQ-C30, the Baduanjin group scored significantly higher than the control group (mean difference = 10.57, 95% CI [7.82, 13.32]). The Pittsburgh Sleep Quality Index (PSQI) score for sleep quality of the Baduanjin group is significantly lower than the control group (mean difference = −2.89, 95% CI [−3.48, −2.30]).

**Conclusion:**

In conclusion, we found the Baduanjin exercise had positive clinical effects on cancer patients. This meta-analysis not only supported that the Baduanjin exercise can alleviate the degree of cancer-related fatigue in patients but also improved their quality of life and sleep quality. Further long-term follow-up randomized controlled trials are warranted.

## 1. Introduction

Nowadays, cancer patients live longer due to advances in cancer treatments. However, a large proportion of cancer patients could suffer from multiple physical and psychosocial complications, such as cancer-related fatigue, deterioration in quality of life (QoL), and sleep quality caused by cancer and its current therapy [1–3]. These negative conseruences may persist for months or years after the therapy [4, 5]. Therefore, more cancer care services should be developed to address the prevention of complications and assist patients in returning to the health status after treatment.

Physical activity is a potentially beneficial intervention to improve survival and QoL for people with cancer [6]. Findings from most studies have reported that exercise is a solution to pain, fatigue, and physical and mental function [7–10]. There is further evidence concerning the benefits of aerobic exercise in the rehabilitation of patients following cancer treatments [11]. A recent meta-analysis summarized that cancer patients in the aerobic training group reduced cancer-related fatigue compared to patients who received routine care [12]. Thus, aerobic exercise was suggested to be included in a part of cancer care and considered as an adjunct therapy that assists in alleviating the negative effects of cancer and its treatment [13].

Qigong is a general term for various exercises and treatments that Chinese people have performed for health [14]. Traditional Chinese medicine believes that Qigong can refine one's life and integrate mind and body [15]. Baduanjin, a branch of traditional Qigong exercise is a mild-to-moderate intensity aerobic exercise, which consists of eight effortless movements that work on specific body parts and meridians [16]. Since Baduajin is a mild and safe aerobic exercise, it is appropriate for cancer patients. Baduanjin may be a potential solution to complications undergoing cancer treatments. This present study was to examine the clinical effects of the Baduanjin Qigong exercise among cancer patients.

## 2. Methods

### 2.1. Literature Search and Search Strategy

We conducted a systematic review and meta-analysis using randomized controlled trials (RCTs) to assess the effects of the Baduanjin Qigong exercise on cancer patients. We searched Cochrane Library, PubMed, Embase, and Airiti Library for all relevant studies from inception through December 31, 2020, without language limitations. The search string used the following: (cancer OR tumor OR tumour OR carcinoma OR neoplas*∗* OR malignan*∗*)) AND (Baduanjin) AND (fatigue OR tiredness OR quality of life OR QOL OR activities of daily living OR ADL OR sleep quality OR sleep disorder OR efficacy OR effect OR effectiveness) (Table 1). We further identified other similar studies through checking the references and similar articles of screening studies.

### 2.2. Study Selection

These studies were involved if they met the following inclusion criteria: (1) the study design was an RCT; (2) they were original studies; (3) both experimental group and control group were diagnosed with cancer; and (4) Baduanjin Qigong exercise was the main intervention for the experimental group. The full texts of the search results were checked carefully, and we obtained the studies which had met the criteria mentioned before. Two authors (Chi-Chun Kuo and Chiao-Chen Wang) independently screened selected studies, evaluated the quality of included studies, and extracted information. Any disagreement was solved through discussion with the third author (Tao-Hsin Tung).

### 2.3. Data Extraction and Assessment of Potential Bias

A data extraction form was used to summarize the following information from selected studies: first author, publication year, country, study period, assigned group, randomly assigned participants (n), types of any participants, intervention time, outcome, and its measurement. An assessment of potential bias was performed independently by the authors. Two authors also used the Cochrane Collaboration Tool to assess the risk of bias of the included trials by the Review Manager version 5.3. The risk assessment tool included the following seven domains of bias risk: (1) random sequence generation, (2) allocation concealment, (3) blinding of participants and personnel, (4) blinding of outcome assessment, (5) incomplete outcome data, (6) selective reporting, and (7) other sources of bias. In the same way, any disagreement was determined to the third author.

### 2.4. Statistical Analysis

We used the Review Manager version 5.3 (The Nordic Cochrane Centre, The Cochrane Collaboration, 2014) to perform the quantitative synthesis. The outcomes of intervention effect were defined using questionnaires at baseline and after all interventions. The intervention effect was summarized by using the odds ratio with 95% CI or mean difference with 95% CI. If the data about means, standard deviation, or *P* value was unclearly reported in the articles, we would calculate by calculator tool in Review Manager or consult the Cochrane Handbook. In addition, we evaluated heterogeneity by using the *I*^2^ statistic. A *P* value <0.10 was considered significantly heterogeneous. Heterogeneity was stratified as undisturbed (*I*^2^: 0–24.9%), low (*I*^2^: 25–49.9%), moderate (*I*^2^: 50–74.9%), or high (*I*^2^: 75–100%). We used the fixed-effect model when *I*^2^ was less than 50% and would have used the random effects model if *I*^2^ was 50% or more. For analyzing MD and 95% CI, if the standard deviation (SD) was not reported, we estimated SD by T-value and standard error. A funnel plot was used to test the symmetry of the pooled results and evaluated the publication bias of meta-analysis.

### 2.5. Critical Appraisal

We assessed the certainty in evidence from our three primary outcomes by using the Grading of Recommendations Assessment, Development and Evaluation (short GRADE). This system critical appraisal considered five domains: (1) overall risk of bias (randomization, allocation concealment, blinding, incomplete outcome data, selective reporting), (2) inconsistency (wide 95% CI), (3) indirectness (an indirect comparison of population, outcome, or intervention), (4) imprecision (I^2^ cutoff of 50%), and (5) publication bias (commercially funding sources or study without “negative” findings). We evaluate each category rate as none, not serious, serious, or very serious; we rated the quality of evidence as very low, low, moderate, or high [17].

## 3. Results

### 3.1. Literature Search and Studies Characteristics

**Figure 1:**
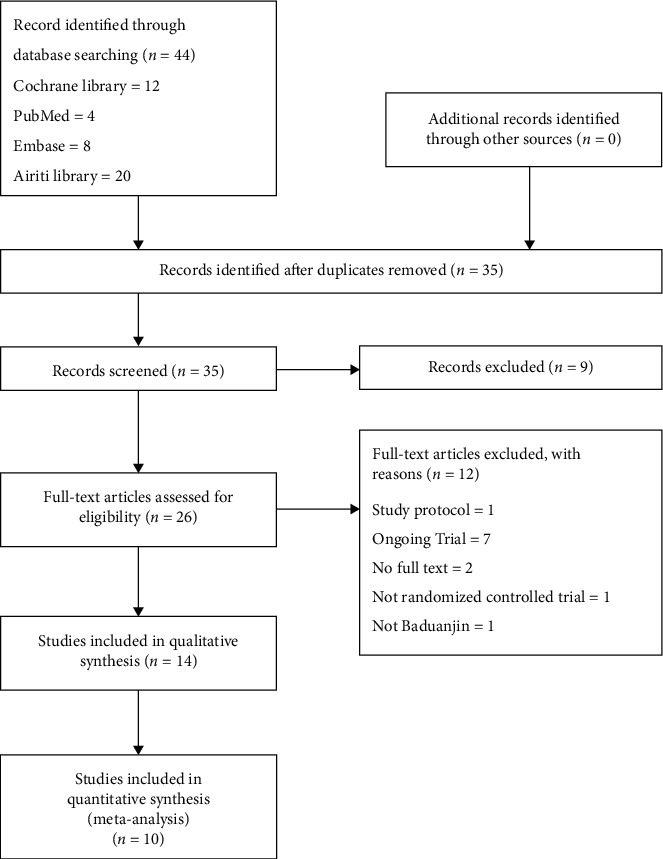
PRISMA (Preferred Reporting Items for Systematic Reviews and Meta-Analyses) flow chart.

**Figure 2:**
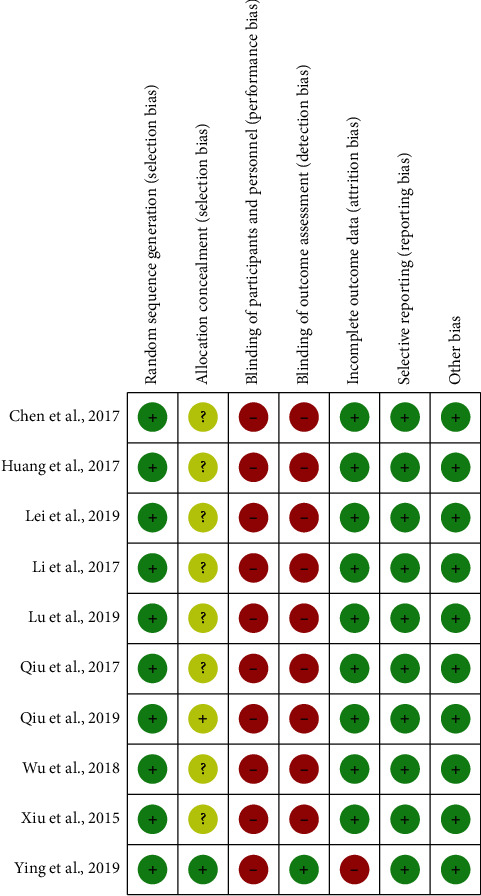
Risk of bias summary.

Figure 1 illustrates the overall study identification process by the Preferred Reporting Items for Systematic Reviews and Meta-Analyses (PRISMA) guidelines. We acquired 44 studies from Cochrane Library, PubMed, Embase, and Airiti Library, of which 10 studies addressed the clinical effects of Baduanjin Qigong exercise on cancer patients. The characteristics of each included study are listed in Table 2. Among these studies, publication years were between 2015 and 2019, and all ten studies were conducted in China. A total of 409 participants were included in the intervention group and 402 in the controls.  Figure 2 presents a summary assessment of bias risk by the Cochrane Collaboration Tool. Ying et al. clearly described how the researcher prevents selection bias by concealing the allocation sequence. The other ten trials did not clearly depict how research populations are selected. Almost all ten trials did not clearly illustrate whether the participants and outcome assessments were blinded since it was not feasible to blind the participants and Baduanjin Qigong exercise coach.

### 3.2. Cancer-Related Fatigue from BFI Measurement

**Figure 3:**
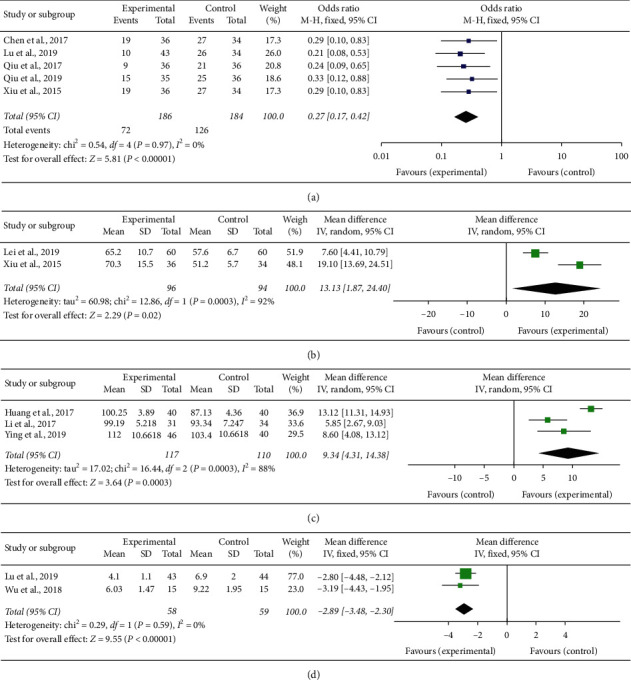
(a) Meta-analysis result of BFI of cancer-related fatigue. (b) Meta-analysis result of EORTC QLQ-C30 of quality of life. (c) Meta-analysis result of FACT-B of quality of life. (d) Meta-analysis result of PSQI of sleep quality.

Five of the studies examined the effect of Baduanjin Qigong exercise on cancer-related fatigue. They provided events of data on this outcome. We organized data from the selected trials using fixed models because of low heterogeneity (*χ*^2^ = 0.54, *P*=0.97, and *I*^2^ = 0%). We found cancer patients with moderate-severe cancer-related fatigue were significantly less in the Baduanjin group compared with the control group (odds ratio = 0.27; 95% CI [0.17, 0.42]) and the test for overall effect presented (*Z* = 5.81, *P* < 0.00001) (Figure 3(a)).

### 3.3. QoL from EORTC QLQ-C30 Measurement

QoL was assessed on the basis of the score for various domains from the EORTC QLQ-C30. The MD was 10.57 (95% CI [7.82, 13.32]). We organized data from the selected trials using random models because of high heterogeneity. Statistical heterogeneity was observed across the trials (*Z* = 7.53, *P* < 0.00001; *χ*2 = 12.86, *P*=0.0003, *I*^2^ = 92%) (Figure 3(b)).

### 3.4. QoL from FACT-B Measurement

Also, three studies showed that Baduanjin Qigong exercise has a positive effect on breast cancer patients. The pooled MD was 11.04 (95% CI [9.56, 12.53]), test for overall effect presented (*Z* = 14.57, *P* < 0.00001; *χ*2 = 16.44, *P*=0.0003, and *I*^2^ = 88%) (Figure 3(c)).

### 3.5. Sleep Quality from PSQI Measurement

Two of the studies focused on the effect of Baduanjin Qigong exercise on the sleep quality of patients with cancer. For the PSQI questionnaire, the MD was −2.89 (95% CI [−3.48, −2.30]), test for overall effect presented (*Z* = 9.55, *P* < 0.001; *χ*^2^ = 0.29%, *P*=0.59, and *I*^2^ = 0%) (Figure 3(d)).

### 3.6. Publication Bias

**Figure 4:**
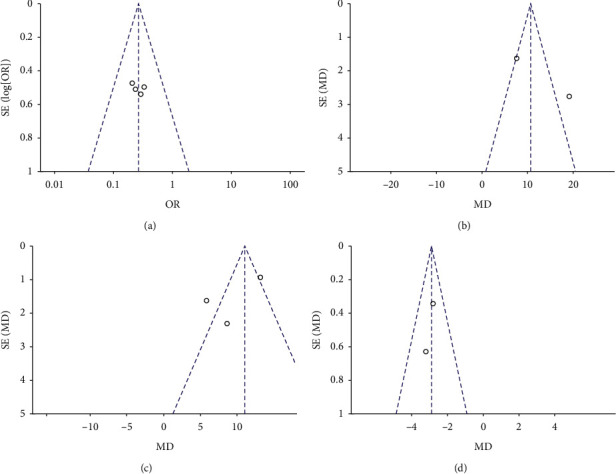
(a) Funnel plot of BFI of cancer-related fatigue. (b) Funnel plot of EORTC QLQ-C30 of quality of life. (c) Funnel plot of FACT-B of quality of life. (d) Funnel plot of PSQI of sleep quality.

Publication bias was viewed as the publication or nonpublication of studies relying on the direction and statistical significance of the results and the first systematic investigations of publication bias focused on this aspect of the problem. As Figures 4(b) and 4(c) show, the funnel plot was asymmetry, indicating some publication bias in this study.

### 3.7. Quality of Evidence Assessment

A detailed evidence assessment of study outcomes is available in Table 3. There was a low quality of evidence for the outcomes of fatigue and sleep quality due to lack of blinding the participants and outcome assessments, as well as allocation concealment; however, in the aspect of blinding, it is impractical to blind the Baduanjin intervention group and their instructors. The quality of evidence for QoL graded very low as a result of not only bias risk but also inconsistency.

## 4. Discussion

### 4.1. Clinical Implications

To the best of our knowledge, this study is the first systematic review and meta-analysis to examine the clinical effects of the Baduanjin Qigong exercise on cancer patients. There is consensus among researchers regarding the Baduanjin Qigong exercise as a potential solution to the complications of cancer and its therapy. Our results supported that Baduanjin Qigong exercise can have a positive impact on cancer-related fatigue, QoL, and sleep quality of patients with cancer. From the clinical viewpoint, common complications occurring during or after cancer treatment and lack of a gold standard for the treatment of complication are problems which should be noticed that many academic studies were conducted to find appropriate health care. After completing this systematic review and meta-analysis, we acquired an interesting outcome that continuous Baduanjin Qigong exercise might have positive effects on cancer patients.

### 4.2. Clinical Practice

Nonpharmaceutical treatments have experienced the stage of development, examination, and implementation. Among them, traditional Chinese medicine's nonpharmaceutical exercise treatments are constantly used for chronic health conditions in China [28, 29]. Low-intensity exercise therapies such as Tai Chi Chuan, Qigong, and Baduanjin are recommended for cancer patients and survivors, especially the easy at-home exercise program [30, 31]. Qigong exercise system like Baduanjin involves a series of movements, breath control, and meditation, which could enhance and strengthen the function of all the internal organs and bodily systems [32]. Thus, compared to traditional routine care for cancer patients suffering from complications, there is a good deal of advantages using the Baduanjin Qigong exercise as an adjunctive treatment for cancer patients.

### 4.3. Heterogeneity of Meta-Analysis

In the meta-analysis, heterogeneity may exist if the sample estimates for the population risk were of different magnitudes [33]. The *I*^2^ statistic implies the percentage of variation across selected studies that is due to heterogeneity rather than chance. In this study, we used the random effect model when *I*^2^ statistics were 92% and 88% for EORTC QLQ-C30 and FACT-B of quality of life more than 50%, respectively. For the existence of significant heterogeneity in QoL, it is important to assess heterogeneity in the meta-analysis. This problem could be caused by the fact that there were only two and three studies included in the groups, respectively, which have a kind of different effect (different level of preference in the experimental group). We aggregate studies that are different methodologies; heterogeneity in the results is still inevitable.

### 4.4. Methodological Considerations

Using meta-analysis study design could not only combine and synthesize multiple studies and integrate the findings, but also conduct research by combining selected studies and providing a precise estimate of the outcome [34]. However, there were some limitations in the present study. Firstly, due to fact that the number of selected studies which could be searched was insufficient, the relative lower statistical power with smaller sample sizes is not avoidable. Secondly, it is controversial of surrounding random effects model. The statistical assumption of the normal distribution for random effects does not fit the principle of randomization [35]. The variance of random effects would be only as an encumbrance variable if there are no random effects. The statistical inference of this nuisance variable to meta-analysis weights would then be to significantly increase variance estimated and consistent weights through exacting the larger studies [36]. Thirdly, most of the studies lack blinding, which may cause performance and detection bias. The biases are inevasible because the Baduanjin intervention group knows what treatment they involve. Fourthly, the usage of different outcome measurements in different studies resulted in only a few studies being included in the analysis, so the strength of the conclusions may be called into question. Fifthly, clinically, the Baduanjin intervention should be conducted on the basis of routine rehabilitation. Therefore, it is difficult to suppose whether the clinical effects were due to Baduanjin alone. Sixthly, the intervention time varies among the finding studies, lasting from 4 weeks to 6 months, and we proposed that long-term effects of the exercise at a certain level need longer follow-up periods. Fifthly, all ten studies included in this meta-analysis were performance in China and the main study populations were Chinese. Therefore, whether the result can extrapolate to non-Chinese population needs further investigation. Finally, only patients of specific age and health conditions participated in these studies, which makes the effects of the Baduanjin Qigong exercise on participants with different characteristics hard to be concluded.

## 5. Conclusions

In conclusion, the current evidence supports that Baduanjin Qigong Exercise has positive clinical effects on cancer patients. This meta-analysis supported that the Baduanjin exercise not only can alleviate the degree of cancer-related fatigue in patients but also can improve their QoL and sleep quality. However, most of the selected studies do not mention the quality control of the practice of the Baduanjin Qigong Exercise. It is difficult to evaluate truly the practice of the Baduanjin Qigong Exercise and effectively estimate the level of each practitioner's practice for the explanation of its actual effect. If this question is not evaluated, and the comparison with the control group has no practical significance, most studies do not consider this issue, so the reliability of the conclusions reached is limited. Further long-term follow-up randomized controlled trials consider the practice of Baduanjin Qigong Exercise's action, breathing, and mind all have the problem of whether the operation in its place would make the research more discursive.

## Figures and Tables

**Table 1:** Search strategy.

1	Cancer
2	Tumor
3	Tumour
4	Carcinoma
5	Neoplas^*∗*^
6	Malignan^*∗*^
7	1 OR 2 OR 3 OR 4 OR 5 OR 6
8	Baduanjin
9	7 AND 8
10	Fatigue
11	Tiredness
12	Quality of life
13	QOL
14	Activities of daily living
15	ADL
16	Sleep quality
17	Insomnia
18	Sleep disorder
19	10 OR 11 OR 12 OR 13 OR 14 OR 15 OR 16 OR 17 OR 18
20	Efficacy
21	Effect
22	Effectiveness
23	20 OR 21 OR 22
24	19 OR 23
25	7 AND 8 AND 24

**Table 2:** Characteristics of randomized controlled trials.

Author	Publication year	Country	Study period	Assigned group	Randomly assigned participants (*n*)	Types of any participants	Intervention time	Outcome
Lu et al. [18]	2019	China	Unclear	Control: routine care	44	Colorectal cancer patient with cancer-related fatigue	24 weeks	Cancer-related fatigue (BFI): (i) At 12 weeks, case (58.1%) vs. control (61.4%); *P*=0.750. (ii) At 24 weeks, case (23.2%) vs. control (59.1%); *P* < 0.01
				Experimental: Baduanjin on the basis of the control group	43			Sleep quality (PSQI): (i) At 12 weeks, case (5.7 ± 1.3) vs. control (7.7 ± 2.0); *P* < 0.01. (ii) At 24 weeks, case (4.1 ± 1.1) vs. control (6.9 ± 2.0); *P* < 0.01

Ying et al. [19]	2019	China	Unclear	Control: original physical activity	40	Breast cancer survivor	6 months	Quality of life (FACT-B) case (112.00 y of l) vs. control (103.40 y of l); *P*=0.000
				Experimental: Baduanjin	46			

Lei et al. [20]	2019	China	Unclear	Control: routine nursing	60	Chemotherapy patients with small cell lung cancer	8 weeks	Quality of life (EORTC QLQ-C30): case (65.2 ± 10.7) vs. control (57.6 ± 6.7); *P* < 0.01
				Experimental: Baduanjin on the basis of the control group	60			

Wu et al. [21]	2018	China	Unclear	Control: routine care	15	Chemotherapy patient with colorectal cancer	2 months	Sleep quality (PSQI): case (6.03 ± 1.47) vs. control (9.22 ± 1.95); *P*=0.000
				Experimental: Baduanjin on the basis of the control group	15			

Huang et al. [22]	2017	China	Unclear	Control: routine rehabilitation exercise	40	Chemotherapy patient after breast cancer radical mastectomy	4 months	Quality of life (FACT-B): Case (100.25 ± 3.89) vs. control (87.13 ± 4.36); *P* < 0.01
				Experimental: Baduanjin on the basis of the control group	40			

Li et al. [23]	2017	China	Unclear	Control: routine rehabilitation	30	Radiotherapy patient after breast cancer radical mastectomy	3 months	Quality of life (FACT-B): case (99.19 ± 5.218) vs. control (93.34 ± 7.247)
				Experimental: Baduanjin and routine rehabilitation	31			

Xiu [24]	2015	China	Unclear	Control: routine nursing	34	Patient with cancer-related fatigue	8 weeks	Cancer-related fatigue (BFI): case (52.8%) vs. control (79.4%); *P*=0.019
				Experimental: Baduanjin and routine nursing	36			Quality of life (EORTC QLQ-C30): case (70.3 ± 15.5) vs. control (51.2 ± 5.7); *P*=0.024

Min and Yan [25]	2017	China	Unclear	Control: conventional nursing	34	Cancer-related fatigue in patient with chemotherapy for acute myeloid leukemia	4 weeks	Cancer-related fatigue (BFI): case (52.7%) vs. control (79.4%); *P* < 0.05
				Experimental: Baduanjin combined with five-element musicotherapy on the basis of the control group	36			

Ping et al. [26]	2019	China	Unclear	Control: conventional nursing	36	Patient with malignancies	4 weeks	Cancer-related fatigue (BFI): case (42.86%) vs. control (69.44%); *P*=0.010
				Experimental: Baduanjin and emotional nursing on the basis of the control group	35			

Ping et al. [27]	2017	China	Unclear	Control: conventional nursing	36	Patient with cancer-related fatigue	8 weeks	Cancer-related fatigue (BFI): case (25.0%) vs. control (58.3%); *P* < 0.05
				Experimental: moxibustion combines with Baduanjin exercise on the basis of the control group	36			

**Table 3:** Quality assessment.

Outcome (measurement)	Number of participants (trials)	Study design	Risk of bias	Certainty assessment				Quality of the evidence (GRADE)	Recommendation
				Inconsistency	Indirectness	Imprecision	Publication bias		
Cancer-related fatigue (BFI)	370 (5)	RCTs	Serious	None	None	Not serious	Undefective	⊕⊕⃝⃝ Low quality	Strong for using
Quality of life (EORTC QLQ-C30)	190 (2)	RCTs	Serious	Serious	None	Serious	Undefective	⊕⃝⃝⃝ Very low quality	Strong for using
Quality of life (FACT-B)	227 (3)	RCTs	Serious	Serious	None	Serious	Undefective	⊕⃝⃝⃝ Very low quality	Strong for using
Sleep quality (PSQI)	117 (2)	RCTs	Serious	None	None	Not serious	Undefective	⊕⊕⃝⃝ Low quality	Strong for using
